# Editorial: Visual rehabilitation of mild traumatic brain injuries or concussion

**DOI:** 10.3389/fnins.2026.2021547

**Published:** 2026-10-20

**Authors:** Tiong Peng Yap, Sowjanya Gowrisankaran, Chang Yaramothu

**Affiliations:** 1 IGARD Vision Therapy Center, Singapore, Singapore; 2 Department of Ophthalmology, Casey Eye Institute, Oregon Health and Science University, Portland, OR, United States; 3 School of Applied Engineering and Technology, New Jersey Institute of Technology, Newark, NJ, United States

**Keywords:** concussion, neuro optometric rehabilitation, neurorehabiliation, ocular motor disorder, oculomotor dysfunction, traumatic brain injury (TBI), vision rehabilitation, vision therapy

Mild traumatic brain injury (mTBI), commonly referred to as concussion, affects millions of adults and children worldwide annually (Skandsen et al., 2018; Gardner and Yaffe, 2015). While historically categorized as “mild” due to the absence of gross structural lesions on standard neuroimaging, the persistent symptoms caused by diffuse axonal injury and subtle neural network disruptions can be profoundly disabling to these patients, affecting their quality of life (Inglese et al., 2005). Among the most frequent, yet frequently underdiagnosed, consequence of mTBI is oculomotor dysfunction (also known as ocular motor disorder), which ranges from inaccurate pursuit and saccadic eye movements, fixational deficits, vergence disorders, accommodative dysfunctions, to poor vestibular-visual integration (Yap and Stern, 2024). For this reason, the oculomotor and visual sensory systems of each mTBI patient warrants an independent clinical evaluation, as the full extent of injury to each of these neurological components within this integrated system requires further investigation (Yap and Stern, 2024).

While the intervention strategies vary across healthcare disciplines, (Chen et al., 2025) structured vergence and accommodative therapy (i.e., vision therapy or neuro optometric vision rehabilitation therapy) can effectively remediate post-concussion oculomotor dysfunctions, reduce symptoms, and enhance functional outcomes through neural plasticity (Alvarez et al., 2012). In addition, objective eye tracking assessments provide an accessible means to evaluate the functional status of the oculomotor system and determine the extent of injury (Yaramothu and Alvarez, 2018). With the combination of vision-specific mTBI survey instruments, this can provide a reliable approach for assessing oculomotor function, gauging injury extent, and defining clear treatment endpoints (Iring-Sanchez et al., 2024). As emerging eye-tracking technologies are likely to make visual rehabilitation more accessible to mTBI patients, integrating objective oculomotor assessments into the patient's overall treatment plans will be essential to quantifying baseline deficits and monitoring treatment outcomes (Figure 1).

**Figure 1:**
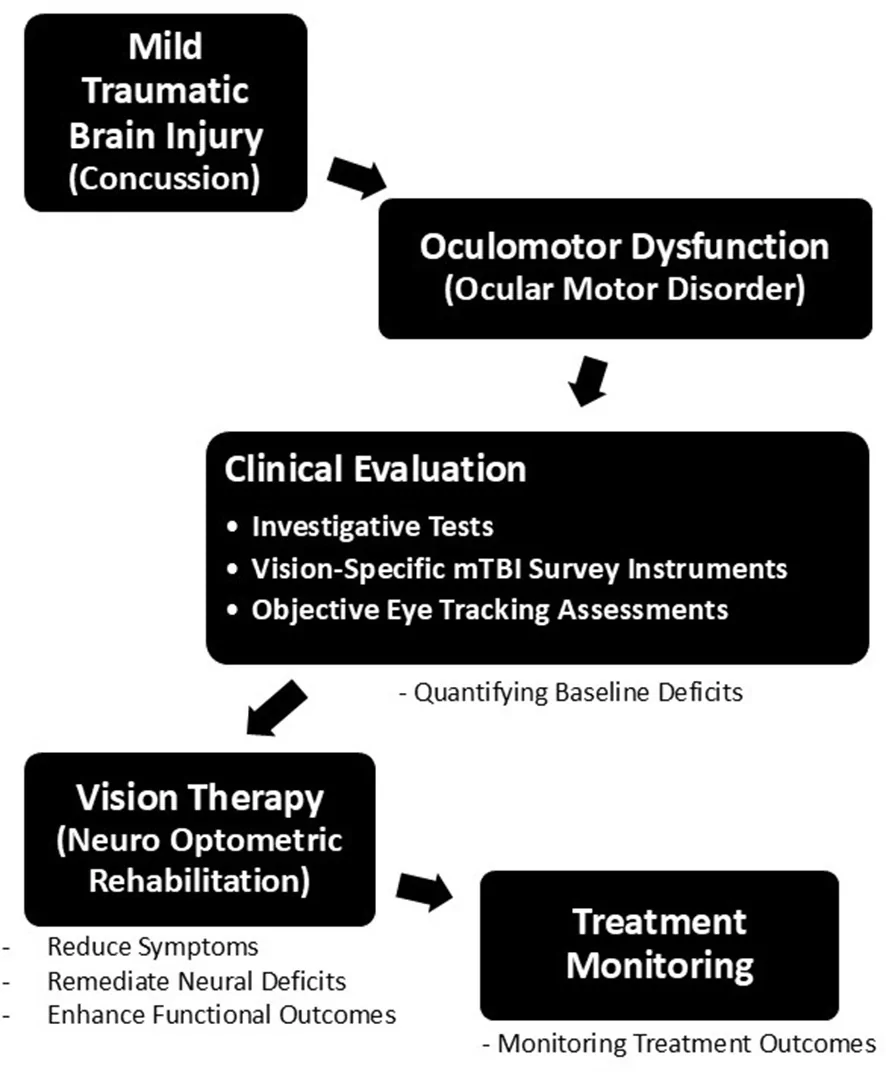
Emerging eye tracking technologies are making vision rehabilitation more accessible to patients with mild traumatic brain injury or concussion.

In this Research Topic on the Visual Rehabilitation of Mild Traumatic Brain Injuries or Concussion, this collection of articles brings together seven original research studies that collectively advance our understanding of objective diagnostic markers, cross-cultural clinical assessment tools and the neural mechanisms underlying neuro visual rehabilitation. Shani et al. evaluated oculomotor markers using simple and predictive visual tracking tasks. Their work demonstrates how subtle alterations in smooth pursuits, saccadic control, and predictive target tracking reflect specific functional impairments following TBI. By quantifying eye tracking errors, oculomotor latencies, pupil responses, and vergence stability, this research provides an objective approach for monitoring recovery and tailoring visual rehabilitation protocols.

While assessing the symptoms of traumatic brain injury (TBI), Almutairi et al. presented a cross-cultural adaptation and validation of the Brain Injury Vision Symptom Survey (BIVSS) into Arabic. Such validated patient-reported outcome measures are crucial to fully capture the nuanced burden of visual symptoms in diverse patient populations. In a complementary study, Almutairi utilized this validated Arabic BIVSS in a case-control evaluation to quantify post-TBI visual symptom burden. The findings highlight the pervasive impact of accommodative dysfunction, light sensitivity and binocular vision disorders on activities of daily living and functioning. Extending these clinical insights to the younger populations, Ghosh et al. investigated the post-concussion symptom profiles using the Convergence Insufficiency Symptom Survey (CISS) within a pediatric and adolescent cohort. Their analysis revealed distinct differences in symptom reporting trends from concussion-related convergence insufficiency, underscoring the need for age-specific diagnostic thresholds and timely intervention to prevent academic and developmental disruptions.

To understand how specific visual rehabilitation and therapy approaches drive neurological recovery, Sangoi et al. reported their functional magnetic resonance imaging (fMRI) findings from the CONCUSS clinical trial, which investigated the neural activity changes following vergence and accommodative therapy in patients with post-concussion convergence insufficiency. Their results demonstrated significant post-rehabilitation alterations in cortical and subcortical activation within the oculomotor network, which provided crucial neurofunctional evidence of activity-dependent neuroplasticity driven by targeted vision therapy. Focusing primarily on symptom modulation and ocular accommodation, Almutairi et al. investigated the effects of colored lenses and neutral density filters on dynamic accommodation in individuals with mTBI. Their findings suggest that the spectral filters can modulate visual pathway gain and provide immediate relief to patients with photophobia while supporting accommodative stability during their visual tasks.

The articles collected in this Research Topic illustrates a crucial paradigm shift in the management of post-concussion oculomotor dysfunction–moving from a reactive management approach toward a more proactive, objective and evidence-based treatment approach in visual rehabilitation. The topic's guest editors, Dr Yap Tiong Peng, Dr Chang Yaramothu, and Dr Sowjanya Gowrisankaran, extends their sincere gratitude to all the authors and peer-reviewers, whose rigorous contributions made this collection a success. The insights gathered from these articles will hopefully inspire more interdisciplinary collaboration among optometrists, ophthalmologists, vision scientists, neurologists, sports medicine, physiotherapists (physical therapists), and neuroscientists. The ultimate goal is to be able to translate these findings into improved functional outcomes and quality-of-life for patients who are recovering from mTBI/concussion.
